# Case of Poisoning with Arsenic, Attended with Remarkable Delay in the Appearance of the Symptoms

**Published:** 1855-12

**Authors:** Edward Hartshorne


### MEDICAL EXAMINER.

NEW SERIES.—NO. CXXXII.—DECEMBER, 1855.

### ORIGINAL COMMUNICATIONS.

### *Case of Poisoning with Arsenic, attended with remarkable delay in the appearance of the symptoms.* By EDWARD HARTSHORNE, M. D.

The following brief sketch of a case of self-inflicted poisoning terminating favorably, is worthy of note, on account of the very unusually long interval that elapsed between the swallowing of at least one drachm, if not two, of arsenious acid, and the appearance of the first decided symptoms of its poisonous action.

I am indebted to the attending physician Dr. Hershey, and to Dr. Griffith, also in attendance after the first day, as well as to the patient herself and to the inmates of the room adjoining hers, for the account of the first four days of her case.

From this it appears that she was a strong, healthy woman, about twenty-two years of age, of a rather excitable nervous temperament, and headstrong impulsive disposition; that she had been subjected for some time previous to great mental agitation, through the threatened withdrawal of an acknowledged admirer, and that under this excitement she had suffered occasional attacks of hysterical spasms. According to her own representation, she had eaten and drunk very little throughout the week ; and on Thursday, the twenty-ninth of March, 1855, she took no food or drink except one cup of coffee at breakfast time. She spent the day in a state of desperation, most of the time on foot, first in seeking an interview with the delinquent lover, and then in procuring from an apothecary, at a long distance from her dwelling place, a paper of arsenic. Her absence from home all day, and her entire abstinence while at home, were testified to by the other inmates of the house. At nine o'clock in the evening of the same day, she had retired to her room, and was heard by the two occupants of the adjoining chamber to be gagging and choking so violently and in such a peculiar manner, that one of them knocked at her door and inquired if there was anything the matter with her. She returned an evasive answer, and remained apparently quiet throughout the night. She kept in bed the next morning, and refused her breakfast, but attracted no special attention until nine o'clock, when the same sound of gagging and choking was heard in her room a second time. In the course of the succeeding two hours an hysterical paroxysm came on. It was then ascertained that she had taken poison, and Dr. Hershey was immediately sent for.

He arrived at 11 o'clock, A. M., just fourteen hours after the first dose had been taken, and two hours after the second. At this stage of the proceedings nothing definite could be learned from her admissions or complaints. She lay in a state of partial cataleptic stupor, occasionally varied with slight muscular spasms such as are common in ordinary hysteria, and seemed to be unwilling or unable to answer questions. The customary effects of irritant poison were so entirely absent, that Dr. Hershey was induced to order an anti-spasmodic potion of some aromatic tincture in camphor water, on the supposition that the attack was a purely nervous one alone. Of this draught she took some four tablespoonfuls, at intervals, in the course of two hours. According to her own repeated assertion, which there is no reason to disbelieve, it was the first fluid which she took into her stomach during at least thirty-six hours—that is for twenty-four hours before and some twelve or thirteen hours after the taking of the evening portion of the arsenic. No change beyond the abatement of the hysterical symptoms occurred until one o'clock in the afternoon, about two hours after she had swallowed the first of the camphor water, when the doctor was hurriedly recalled on account of the sudden onset of violent pain and vomiting. The most frequent, and in this instance, at that time, the only positive, symptoms of arsenical poisoning had at last presented themselves, sixteen hours after the first powder had been swallowed, and four hours after the second.

The fresh hydrated oxide of iron was immediately given and continued, in divided doses, to the amount of five ounces; but notwithstanding this and the free use of sulphate of morphia and cold mucilaginous drinks internally, and of depletion with cups and the subsequent application of cataplasms and a blister externally, the pain and vomiting increased in severity and frequency until the afternoon of Sunday, the third day. She then appeared to be so utterly prostrated that no hopes were entertained of her recovery either by herself or her physicians. A paper containing a portion of the white arsenic of the shops had already been found in her room. The apothecary had been visited, and had confirmed her story as to its purchase on the preceding Thursday. The amount remaining in the packet had been compared with the quantity stated by her to have been swallowed. Questions were again asked her, when she was supposed by all to be in extremis, and were answered as frankly and fully as could be desired. These answers being given under the impression that she was at the point of death, are fairly entitled to belief for that reason alone; but they were corroborated wherever possible by other testimony, and invariably supported by her own statements in answer to the repeated and varied cross-questionings to which she was subjected by her medical attendants during convalescence, and after every inducement for a perversion or suppression of the truth had been removed.

The history thus gathered from the patient and her friends, was, in a few words, that her whole mind had become absorbed in the thought of her troubles, and the desire to end them in the grave; that with this intent, having secretly provided herself with arsenic, and retired to her room after a whole day of fasting and agitation, she attempted to swallow a teaspoonful of the dry powder and was so irritated in the throat by it as to alarm her neighbors. She coughs out a part of it, but manages to retain about half-a-teaspoonful. She lies down, as she expects, to die, but spends the night without any change whatever, and, as she thinks, without sleep. The next morning she swallows another half-teaspoonful in the same manner and with the same difficulty. Soon after this last attempt, she loses her self-control, becomes hysterical, and announces her probable fate to the family who come to her aid. During all this time, however, she feels no nausea, thirst or pain, and has not the slightest idea of the agony she is shortly to endure. It is not until after she has taken several drinks, that she begins to realize the horrors of her situation. Her entire ignorance of the effects of arsenic was fully demonstrated, and of itself afforded satisfactory proof that, although intelligent and tolerably well educated, as well as a determined girl, she could not possibly have simulated her disease, or so contrived to regulate the dose as to effect a partial poisoning only. The symptoms, however, were too well marked in their course and violence to admit of any doubt as to the existence and progress of an irritant poison. Nothing more was required to demonstrate the presence of arsenic, except the chemical examination of the matter thrown up from the stomach, and of the fæces and urine. Unfortunately, this examination was not made. My attention was first called to the case on the evening of the third day, Sunday. The vomiting and pain had then ceased. Reaction had begun, although the patient was still in a partial collapse, with feeble, quick pulse, pale sunken countenance, cool, moist skin, great muscular debility, and a tendency to hebetude with occasional temporary cataleptic spasms. The pharyngitis, tympanitis and epigastric and hypogastric tenderness were very well marked. These gradually subsided, and were accompanied and succeeded by similar evidences of inflammation running along the course of the colon down to the rectum, and going off with tormina, tenesmus, bloody stools, hemorrhoidal irritation and strangury, and followed for a few days by an acne-like eruption of the skin.

The treatment, which was demulcent, anodyne and cautiously supporting, with a gradually improving regimen, need not be particularly detailed. The youth and general health of our patient secured her a comparatively rapid convalescence. By the end of five or six weeks, she had recovered her former flesh and color, and appeared to have escaped with unimpaired gastric and intestinal functions. The only difficulty that remained, was a debility of the lower extremities, amounting to a partial paraplegia, and a relaxed condition of the pelvic organs, which led, on two different occasions, to a painful retroversion of the uterus. Both of these infirmities, however, were so far removed in the course of another month, that, at the latter part of June —within three months after her attempt—she considered herself perfectly restored to health.

It was of course impossible to determine the amount of arsenic actually swallowed in this instance. A sufficiently near estimate, however, was reached through the patient's own statement, made under circumstances that afforded no inducement for deception, and confirmed to a considerable extent by her fellow-lodgers, and by the apothecary who furnished the packet from which the two portions must have been taken.

Supposing her to have retained no more than three quarters of a teaspoonful altogether, which is the smallest probable amount, she must, according to the approximative measurements given by Taylor, have been exposed to the destructive action of at least one hundred and twelve grains in all, and to that of fifty-six of these grains, thirteen or fourteen hours before the appearance of any nervous symptoms, and just sixteen hours before the accession of pain, vomiting and thirst.

The cerebro-spinal demonstrations presented nothing sufficiently different from those of ordinary hysteria to warrant their being regarded as the narcotism sometimes produced by arsenical intoxication. Still, allowing these disturbances to have been the premonitory signs of more serious poisonous action, we yet have an interval of fully thirteen hours' duration, after the ingestion of nearly a drachm of the acid without the advent of a single irregular sensation.

Cases are reported in which arsenic has begun its work at once upon its victims. More frequently, a few minutes have elapsed; but the average period at which the deleterious operation generally begins to manifest itself, is stated to be from half an hour to an hour. Taylor refers to a case communicated to him by Mr. Todd, in which no symptoms appeared for two hours, after one drachm had been taken on an empty stomach. Orfila relates one in which five hours passed, after more than half an ounce had been taken, without decided mischievous effect; Devergie reports an interval of four hours; Taylor quotes another of nine hours duration, and one from Belloc, (see Taylor On Poisons, p. 259,) in which the symptoms did not present themselves for ten hours. This last is the longest instance of uncomplicated freedom from noxious symptoms hitherto on record. There are others besides these, of different periods of protraction, which need not be detailed. The case of Tonnelier, (see Orfila, Toxicologie, 1, 386,) although very remarkable, is not entirely free from doubt, as an instance of retarded progress in arsenical poisoning. That of Clegg, also, (see Taylor, Med. Jurisp. 76,) can hardly be included in the class, because it must have been affected by the modifying influence of opium upon the arsenical irritation. I have not been able to find, in the Journals, any analogous cases not already cited in the works on medical jurisprudence; and I am, therefore, inclined to believe that our patient stands alone in her experience, so far at least as the published annals of toxicology have gone.

The bearing of such experience on the medical evidence in certain criminal cases, may be easily understood. Christison expressly recognizes the importance of it in his work on Poisons, (p. 237,) and refers to a trial, in which a woman escaped conviction, principally because "the symptoms of poisoning did not begin until more than eight hours after, the only occasion on which the prisoner was proved to have administered anything in a suspicious manner." "As I was not at that time," continued he, "acquainted with any parallel case, except that recorded by Orfila, I hesitated to ascribe the symptoms to the draught; and, consequently, as the other medical witnesses felt the same hesitation on the same account, the proof of administration was considered to have failed. I am not sure that I should have now felt the same difficulty."

Our case seems to me to possess considerable interest, also, in a physiological and pathological point of view; but as my object is especially to report the observation for what it may be worth in questions of medical evidence, I must leave to the ingenuity of other inquirers, all speculations as to the cause and nature of the delay of gastric disorder under the peculiar circumstances stated.

